# Dihydromyricetin inhibits African swine fever virus replication by downregulating toll-like receptor 4-dependent pyroptosis in vitro

**DOI:** 10.1186/s13567-023-01184-8

**Published:** 2023-07-12

**Authors:** Yang Chen, Zebu Song, Hao Chang, Yanchen Guo, Zhi Wei, Yankuo Sun, Lang Gong, Zezhong Zheng, Guihong Zhang

**Affiliations:** 1 Guangdong Provincial Key Laboratory of Zoonosis Prevention and Control, College of Veterinary Medicine, South China Agricultural University, Guangzhou, China; 2 African Swine Fever Regional Laboratory of China (Guangzhou), Guangzhou, China; 3 Research Center for African Swine Fever Prevention and Control, South China Agricultural University, Guangzhou, China; 4 Maoming Branch, Guangdong Laboratory for Lingnan Modern Agriculture, Maoming, Guangdong China; 5 Key Laboratory of Animal Vaccine Development, Ministry of Agriculture and Rural Affairs, Guangzhou, China; 6 National Engineering Research Center for Breeding Swine Industry, South China Agricultural University, Guangzhou, China

**Keywords:** African swine fever virus, dihydromyricetin, TLR4/MyD88/MAPK/NF-κB signaling, pyroptosis

## Abstract

African swine fever (ASF), caused by ASF virus (ASFV) infection, poses a huge threat to the pork industry owing to ineffective preventive and control measures. Hence, there is an urgent need to develop strategies, including antiviral drugs targeting ASFV, for preventing ASFV spread. This study aimed to identify novel compounds with anti-ASFV activity. To this end, we screened a small chemical library of 102 compounds, among which the natural flavonoid dihydromyricetin (DHM) exhibited the most potent anti-ASFV activity. DHM treatment inhibited ASFV replication in a dose- and time-dependent manner. Furthermore, it inhibited porcine reproductive and respiratory syndrome virus and swine influenza virus replication, which suggested that DHM exerts broad-spectrum antiviral effects. Mechanistically, DHM treatment inhibited ASFV replication in various ways in the time-to-addition assay, including pre-, co-, and post-treatment. Moreover, DHM treatment reduced the levels of ASFV-induced inflammatory mediators by regulating the TLR4/MyD88/MAPK/NF-κB signaling pathway. Meanwhile, DHM treatment reduced the ASFV-induced accumulation of reactive oxygen species, further minimizing pyroptosis by inhibiting the ASFV-induced NLRP3 inflammasome activation. Interestingly, the effects of DHM on ASFV were partly reversed by treatment with polyphyllin VI (a pyroptosis agonist) and RS 09 TFA (a TLR4 agonist), suggesting that DHM inhibits pyroptosis by regulating TLR4 signaling. Furthermore, targeting TLR4 with resatorvid (a specific inhibitor of TLR4) and small interfering RNA against TLR4 impaired ASFV replication. Taken together, these results reveal the anti-ASFV activity of DHM and the underlying mechanism of action, providing a potential compound for developing antiviral drugs targeting ASFV.

**Supplementary Information:**

The online version contains supplementary material available at 10.1186/s13567-023-01184-8.

## Introduction

African swine fever (ASF) is a highly contagious disease of swine that results in serious economic losses in global pig production. It is caused by the ASF virus (ASFV), the sole member of the *Asfarviridae* family. ASFV is a large, double-stranded DNA virus with a 170–190 kb long genome [1]. ASF is clinically characterized by hemorrhagic fever and nearly a 100% mortality rate in pigs of all ages [2]. It originated in Kenya in 1921 and remains endemic in Africa [3]. In 2018, the first case of ASF was identified in China and spread rapidly to neighboring countries owing to the lack of effective control measures. To date, over 37 countries have reported ASF outbreaks, and although scientists have worked to develop a vaccine against ASFV, and ASFV-G-ΔI177L/ΔLVR, BA71ΔCD2, and the naturally attenuated Lv17/WB/Rie1 are reported to be very promising vaccines, no anti-ASFV drugs or vaccines have been approved to control their spread [4, 5]. Hence, developing new effective measures to reduce the economic losses caused by ASFV and recover pig production is urgently required.

During viral infection, virus-host interactions determine the efficiency of virus propagation, and viruses enhance their own replication by activating cellular signaling pathways. For example, ASFV infection has been reported to activate an ATR-mediated (ataxia telangiectasia mutated rad-3 related) DNA damage response, leading to morphological and functional alterations in subnuclear structural domains, thereby promoting viral replication [6–8]. Therefore, blocking the cellular signaling cascades required for viral replication is an alternative approach to inhibit viral growth. The advantage of targeting the signaling cascades required for viral replication is that resistance is unlikely to develop [9]. The toll-like receptor (TLR) family is a major pattern recognition receptor (PRR) family that recognizes a wide range of bacteria and viruses, and the protein TLR4 is involved in the induction of host immune responses to bacterial, fungal, and viral infections. The TLR4 signaling pathway is divided into two parts, MyD88-dependent and TRIF-dependent signaling. The TLR4-MyD88 signaling axis activates NF-κB and MAPKs and induces the secretion of pro-inflammatory cytokine. The TLR4-TRIF axis promotes the production of interferons and increases the expression of chemokines [10]. However, dysregulated TLR4 signaling has been shown to play roles in the initiation and progression of various diseases, such as sepsis, endotoxemia, acute lung injury, rheumatoid arthritis, and cardiovascular diseases [11]. Viral infection reportedly induces the expression of TLR4; Zhao et al. found that TLR4 interacts with the SARS-CoV-2 spike protein and is activated and that resatorvid, an inhibitor of TLR4, completely blocks the induction of IL-1β by SARS-CoV-2 [12]. In addition, influenza A virus (IAV) can activate TLR4/NF-κB signaling pathway expression in vivo and in vitro, whereas the traditional Chinese medicine Liu Shen Wan inhibits IAV replication by suppressing the expression of TLR4 and phosphorylated NF-κB in vivo and in vitro [13]. Similarly, oxymatrine inhibits IAV replication by suppressing the TLR4, P38 MAPK, and NF-κB pathways and inhibits the inflammation induced by IAV in vivo [14]. These observations indicate that TLR4 may be a target for treating viral infections.

Infection with virulent ASFV isolates induces an increase in serum pro-inflammatory cytokines, resulting in an exacerbated immune response and high mortality; infection with moderately virulent ASFV isolates results in low mortality, which may be associated with the induction of low serum pro-inflammatory cytokines [15]. ASFV infection has been reported to induce upregulation of pro-inflammatory cytokines, including IL-6, TNF-a, CCL4, CXCL8, etc. In addition, TLR3 and TLR7 were upregulated after ASFV infection, whereas TLR4 and TLR6 were downregulated [16]. Another study reported that ASFV I329L inhibits the activation of TLR3, TLR4, TLR5, TLR8, and TLR9 [17]. Moreover, Li et al. showed that the ASFV-induced IL-1β production is dependent on TLR4 [18]; however, the role of TLR4 signaling in ASFV infection has not been reported.

Owing to the complex viral structure and limited cell tropism of ASFV, vaccines against the ASFV are yet to be developed [19]; nevertheless, it is necessary to develop anti-ASFV drugs before an anti-ASFV vaccine can be approved for use. Currently, compounds with anti-ASFV properties include nucleoside analogs, interferons, HDACi (histone deacetylases inhibitors), antibiotics, microtubule-stabilizing agent, and natural compounds [20–23]. Increasing research has proved that natural compounds are widely used in antiviral research because of their broad bioactivity and sources. Toosendanin, genistein, and kaempferol have been reported to inhibit ASFV replication in vitro [24–26]. The natural flavonoid dihydromyricetin (DHM) is derived from *Ampelopsis grossedentata*, exerts anti-inflammatory, anti-bacterial, and anti-tumor activities, and has great potential for clinical application [27, 28]. For example, DHM treatment reduces the activation of NLRP3 inflammasome and lipopolysaccharide (LPS)-induced TLR4/NF-κB signaling pathway [29]. Additionally, DHM exerts antiviral effects and was recently reported as an inhibitor of SARS-CoV-2 M^pro^; thus, it may be a candidate for SARS-CoV-2 infection treatment [30]. However, there are no reports on the use of DHM for ASF treatment.

This study aimed to screen for compounds with anti-ASFV activity and examine their underlying action mechanisms. Our findings would be helpful in developing strategies for preventing ASF spread.

## Materials and methods

### Cells, viruses, and test compounds

Four-week-old specific pathogen-free healthy piglets were euthanized, and porcine alveolar macrophages (PAMs) were collected and cultured in RPMI-1640 medium supplemented with 10% fetal bovine serum, penicillin, streptomycin (100 IU/mL), and L-glutamine (2 mM). Next, ASFV (GZ201801), swine influenza virus (SIV, H1N1, A/Swine/Guangdong/SS1/2012 (Eurasian avian-like swine H1N1 viruses, GISAID accession numbers EPI_ISL_166533)), and porcine reproductive and respiratory syndrome virus (PRRSV, NADC-30) were inoculated in PAMs, titrated using the hemadsorption (HAD_50_) or TCID_50_ assay, and stored at −80 °C. Most test compounds were obtained from Chengdu Chroma Biotechnology Co., Ltd. (Chengdu, China), and genistein was used as a positive control because of its anti-ASFV activity [25]. Resatorvid, EUK-134, RS 09 TFA, polyphyllin VI, and disulfiram were purchased from MedChemExpress (South Brunswick Township, NJ, USA).

### Cell-counting kit (CCK)-8 analysis

The viability of treated PAMs was assessed with the CCK-8 assay. The PAMs were plated into 96-well cell culture plates, incubated for 6 h for adherence, and then treated with serial dilutions of the compounds. After 48 h, PAMs were incubated with 10 µL CCK-8 at 37 °C. After 1 h, the optical density of the plate was determined by measuring the absorbance at 450 nm using a microplate reader (Thermo Fisher Scientific, Waltham, MA, USA). GraphPad Prism 8.0 (GraphPad Software, San Diego, CA, USA) was used to calculate the compounds’ 50% cytotoxic concentration (CC_50_).

### Indirect immunofluorescence assay (IFA)

Cells grown in 48-well plates were fixed with 4% paraformaldehyde at 25 °C for 10 min and washed thrice with phosphate-buffered saline (PBS). Next, cells were incubated with 0.25% Triton X-100 for 10 min at 25 °C and washed thrice with PBS. Cells were then blocked with 3% bovine serum albumin (BSA) at 37 °C for 1 h and washed thrice with PBS. Treated cells were incubated with an ASFV p30 antibody (1:500) at 4 °C overnight, washed thrice with PBS, and then incubated with Alexa Fluor 568 (1:1000) at 37 °C for 1 h. PAMs were incubated with DAPI for 10 min to stain cell nuclei. Leica DMI 4000 B fluorescence microscope (Leica, Wetzlar, Germany) was used to capture images.

### Antiviral activity assay

To investigate the inhibitory effect of DHM on ASFV, cells were infected with 1 MOI (multiplicity of infection) ASFV solution at 37 ℃. After incubating for 2 h, the unabsorbed virus was removed, and the culture medium containing the DHM was added at a two-fold serial dilution. Finally, the viruses were collected and analyzed with the HAD_50_, quantitative reverse transcription polymerase chain reaction (RT-qPCR), and Western blotting assays.

### Time-of-addition assay

The PAMs were grown in culture plates for 6 h and then infected with 1 MOI ASFV at 37 ℃. The DHM was added before (pre-treatment), during (co-treatment), or after (post-treatment) ASFV infection. For pre-treatment, DHM was added to the cells and discarded after 2 h, and the cells were then infected with 1 MOI ASFV after being thoroughly washed with PBS. The supernatant was discarded after 2 h, and fresh culture medium was added to the cells after washing with PBS. For co-treatment, the ASFV solution and DHM were contemporarily added to the cells. The supernatant was discarded after 2 h, and fresh culture medium was added after washing with PBS. For post-treatment, the cells were incubated with ASFV, the supernatant was discarded after 2 h, and the cells were washed with PBS before adding a fresh culture medium containing the DHM. After 48 h, the plates were collected and analyzed via RT-qPCR and Western blotting.

### Virucidal assay

The ASFV (1 MOI) was mixed with DHM and incubated for 1 h at 37 °C; the mixture was then separated using ultrafiltration tubes. To avoid loss of ASFV particles, 100 nm pore size ultrafiltration centrifuge tubes (Sigma-Aldrich, MA, USA) were used to separate DHM and ASFV. Resuspension of the ASFV in ultrafiltration tubes was followed by incubation with PAMs for 48 h, and samples were then analyzed via Western blotting and RT-qPCR.

### Western blotting

Cells cultured in 12-well plates were lysed using RIPA lysis buffer (Beyotime Biotechnology, Shanghai, China), and a bicinchoninic acid kit (Beyotime Biotechnology) was used to standardize the protein content. SDS-PAGE at 10% or 12.5% was used to separate protein samples. Then, 5% BSA was used to block the nitrocellulose membranes for 1.5 h after sample transfer. Next, the following primary antibodies were added: anti-p30 protein (prepared in our laboratory), anti-Actin (AF0003; Beyotime Biotechnology), anti-TLR4 (293,072; Santa Cruz Biotechnology, Inc., Dallas, TX, USA), anti-MyD88 (TA5195S; Abmart, Berkeley Heights, NJ, USA), anti-p-p65 (3033T; Cell Signaling Technology, Boston, MA, USA), anti-p-P38 (4511T; Cell Signaling Technology [CST], Danvers, MA, USA), anti-p-ERK (4370T; CST), anti-NLRP3 (M035175S; Abmart), anti-ASC (bs-6741R; Bioss, Woburn, MA, USA), anti-caspase-1 (22915-1-AP; Proteintech, Rosemont, IL, USA), anti-GSDMD (20770-1-AP; Proteintech), and anti-GSDMD-N (DF12275; Affinity Biosciences, Cincinnati, OH, USA). After washing thrice with TBST, the membranes were incubated with goat anti-mouse or anti-rabbit second antibodies (LI-COR Biosciences, Lincoln, NE, USA), then imaged with the Tanon-5200 multi-infrared imaging system (Shanghai Tianneng Technology Co., Ltd., Shanghai, China).

### Determination of expression of target gene expression using RT-qPCR

The total RNA from ASFV-infected and uninfected PAMs was extracted using a total RNA rapid extraction kit (Fastagen, Shanghai, China); a reverse transcription kit (TaKaRa, Shiga, Japan) was then used to reverse transcribe all samples into first-strand cDNA. RT-qPCR was conducted using the SYBR qPCR Master Mix (Vazyme, Nanjing, China) and Bio-Rad CFX96 system (Bio-Rad Laboratories, Hercules, CA, USA). The amplification conditions used were an initial denaturation step of 95 °C for 30 s followed by 40 cycles of 95 °C for 10 s and 60 °C for 30 s. The primer sequences used in this study are listed in Table 1.

**Table 1:** **Primer sequences of the target genes.**

Target	Sequence (5′–3′)	Orientation
B646L	ATAGAGATACAGCTCTTCCG	Forward
B646L	GTATGTAAGAGCTGCAGAC	Reverse
GADPH	CCTTCCGTGTCCCTACTGCCAAC	Forward
GADPH	GACGCCTGCTTCACCACCTTCT	Reverse
TLR4	TGTGCGTGTGAACACCAGAC	Forward
TLR4	AGGTGGCGTTCCTGAAACTC	Reverse
MyD88	GGCAGCTGGAACAGACCAA	Forward
MyD88	GGTGCCAGGCAGGACATC	Reverse
IL-18	CGATGAAGACCTGGAATCGG	Forward
IL-18	CATCATGTCCAGGAACACTTCTCTG	Reverse
IL-1β	AAGAGGGACATGGAGAAGCGATTTG	Forward
IL-1β	TTGTTCTGCTTGAGAGGTGCTGATG	Reverse
IL-6	TGCCGGCCTGCTGGATAAGC	Forward
IL-6	TGGCCCTCAGGCTGAACTGC	Reverse
TNF-α	CCAATGGCAGAGTGGGTATG	Forward
TNF-α	TGAAGAGGACCTGGGAGTAG	Reverse
NLRP3	CCTTCAGGCTGATTCAGGAG	Forward
NLRP3	GACTCTTGCCGCTATCCATC	Reverse
ASC	GACAACAAACCAGCACTGCACTTCG	Forward
ASC	ACTGCCTGGTACTGCTCTTCCGT	Reverse
caspase-1	ATCTCACCGCTTCGGACATGGCTAT	Forward
caspase-1	GTATTTCTTCCCACAAATGCCAGCC	Reverse
GSDMD	ATGGCATCAGCCTTTG	Forward
GSDMD	CTAGCAGAGCTGGCTG	Reverse

### Determination of reactive oxygen species (ROS) levels

The PAMs were cultured in 24-well plates for 6 h and inoculated with ASFV (1 MOI) for 2 h. Subsequently, the ASFV solution was discarded, and different concentrations of DHM (25–100 µM) were added for 24 h. After washing thrice with PBS, dichloro-dihydro-fluorescein diacetate (DCFH-DA, 10 µM) solution was added; after 30 min, the cells were washed thrice and imaged using a Leica DMI 4000 B fluorescence microscope.

### Small interfering RNA treatment

The PAMs were grown in 12-well cell culture plates for 6 h. Following the manufacturer’s instructions, the cells were transfected with 50 nM siTLR4 (Table 2) using riboFECT™ CP (RiboBio, Guangzhou, China). After 36 h of incubation, the cells were inoculated with an ASFV solution (1 MOI). The samples were collected after 24 h and analyzed with Western blotting.

**Table 2:** **Target TLR4 sequences of small interfering RNA.**

siRNA name	Sequences (5′ to 3′)
siTLR4-a	GCAAATGCCTCTGTGATTT
siTLR4-b	GCGTGTGAACACCAGACTT

#### Statistical analysis

All data are presented as the mean ± standard deviation of values from three independent experiments. Results were analyzed using Student’s *t*-test or a one-way analysis of variance (ANOVA) with the GraphPad Prism 8.0 software. Statistical significance was set at *P* < 0.05.

## Results

### Screening for compounds with anti-ASFV activity

**Figure 1:**
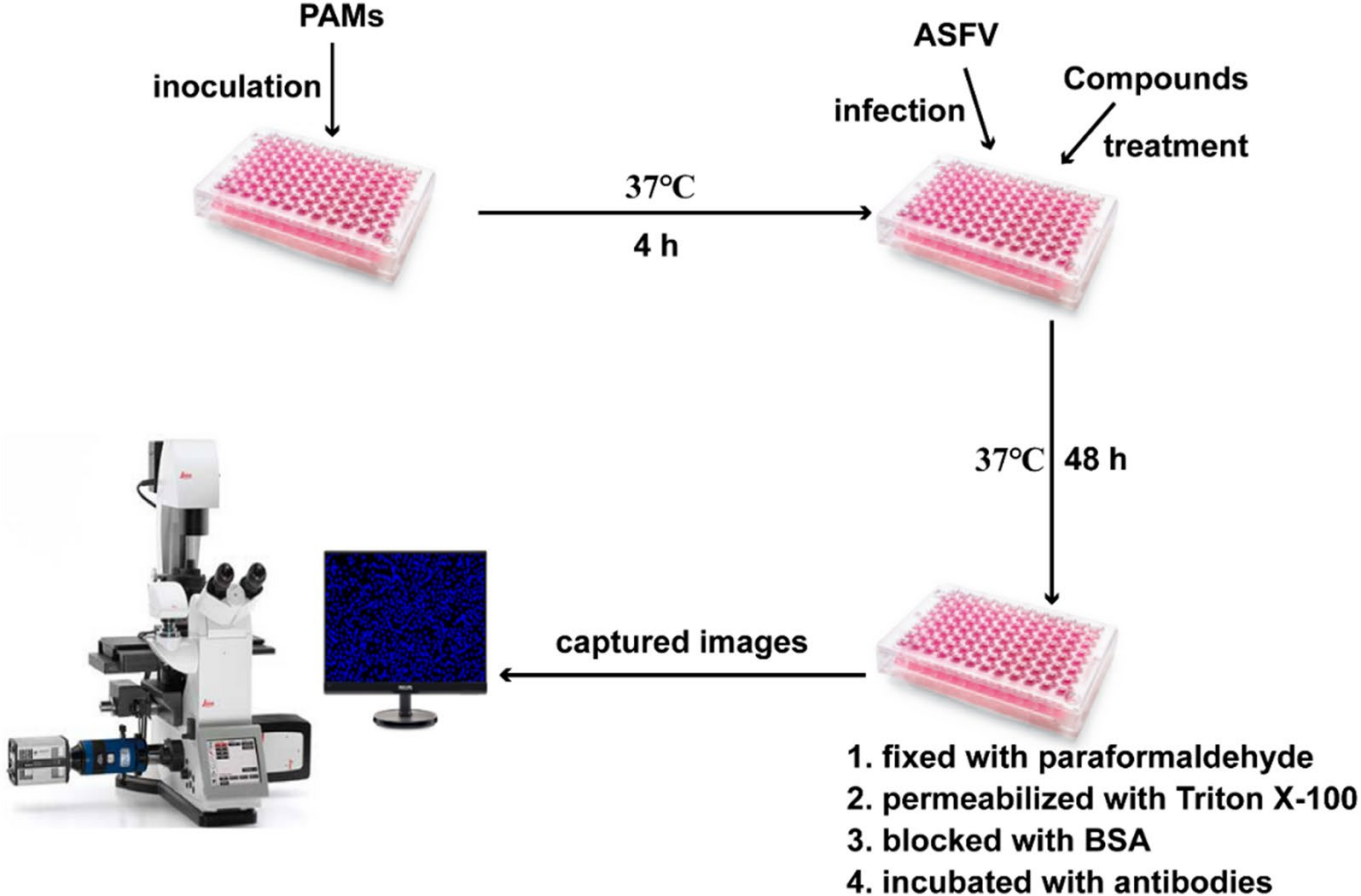
**Diagram of the anti-ASFV compound screening assay design.**

To identify novel ASFV inhibitors, we screened a small chemical library of 102 compounds, most of which were not reported to possess anti-ASFV activity. Their cytotoxicity was tested with the CCK-8 assay. The design of the anti-ASFV activity assay and results are shown in Figure 1; Table 3, respectively. We identified 17 compounds with anti-ASFV activity, including the positive control genistein. The selectivity index (SI) of kaempferol, quercetin, naringenin, formononetin, resveratrol, luteolin, sinensetin, licochalcone A, and DHM was higher than that of genistein (5.59). Moreover, the SI values of kaempferol, quercetin, naringenin, resveratrol, luteolin, and DHM were > 10. Notably, the SI values of DHM (SI = 26.53) were the highest among all tested compounds; therefore, we selected DHM for subsequent experiments.

**Table 3:** **Anti-ASFV activity of the test compounds and their effects on PAM viability.**

Compounds	CC_50_ (µM)	IC_50_ (µM)	SI
Genistein	40.69	7.29	5.59
Kaempferol	115.87	9.55	12.13
Quercetin	336.42	19.38	17.40
Naringenin	595.4	46.98	12.67
Formononetin	600.89	97.77	6.14
Resveratrol	62.41	6.01	10.38
Luteolin	79.11	4.48	17.66
Cinnamaldehyde	531.5	150.6	3.5
Ginsenoside Rb1	229.09	120.3	1.90
Sinensetin	400.43	68.50	5.85
Isofraxidin	692.38	540.29	1.28
Mycophenolic acid	539.4	380.63	1.42
11-Keto-β-boswellic acid	356.7	241.77	1.48
Paeonol	965.87	720.12	1.34
Matrine	106.67	80.63	1.32
Licochalcone A	102.9	10.41	9.88
Dihydromyricetin	532.1	20.06	26.53

CC_50_, 50% cytotoxic concentration; IC_50_, half maximal inhibitory concentration; SI, selectivity index

### DHM treatment suppressed ASFV replication in PAMs

**Figure 2:**
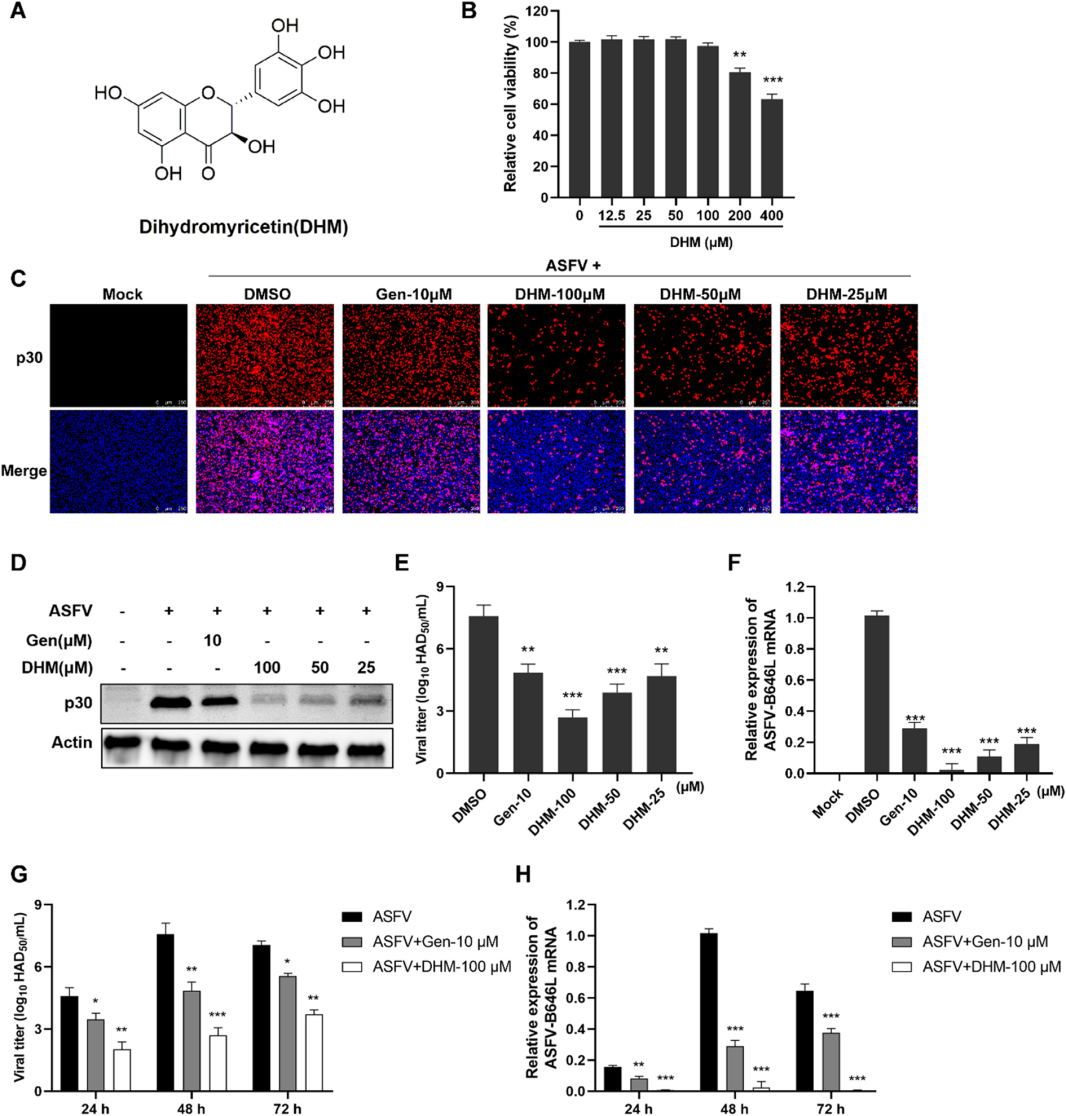
**Anti-ASFV effects of DHM.** **A** Chemical structures of DHM. **B** The viability of DHM-treated PAMs. **C**–**H** The anti-ASFV activity of DHM was evaluated using **C** indirect immunofluorescence assay, **D** Western blotting, **E**, **G** hemadsorption HAD_50_, and **F, H** RT-qPCR. PAMs were attached to the cell plates and incubated with ASFV solution (1 MOI) for 2 h. The supernatant was removed, and cells were replaced with various concentrations of the DHM. The samples were further incubated and collected at 48 h or the indicated time. *P < 0.05, **P < 0.01, and ***P < 0.001 compared to the respective viral control.

To systematically evaluate the anti-ASFV effects of DHM (Figure 2A), we evaluated the antiviral activity of DHM using various assays. First, we evaluated the effects of DHM on PAM viability using the CCK-8 assay and found that 100 µM DHM was non-cytotoxic (Figure 2B). Moreover, IFA and Western blotting assays revealed that at concentrations between 25 and 100 µM, DHM reduced ASFV p30 protein expression levels compared to that in the ASFV control (Figures 2 C and D). We further assessed the effects of DHM on ASFV progeny virus titers and ASFV B646L transcription. The virus titers were reduced in a dose-dependent manner after DHM (25 to 100 µM) treatment, and this reduction was more pronounced at 100 µM, reducing the viral yields from 7.58 to 2.69 log_10_ HAD_50_/mL compared to those in the ASFV control (Figures 2E and F). Similarly, DHM reduced ASFV-B646L transcription in a dose-dependent manner, and 100 µM DHM reduced the expression of ASFV-B646L by approximately 90% compared to that of the ASFV control. Moreover, DHM treatment reduced the ASFV-induced hemadsorption, which is an exceptional phenomenon of the ASFV (Additional file 1).

To investigate whether the reduction effect of DHM on ASFV replication is sustained, we collected samples 24, 48, and 72 h after treatment with DHM and analyzed them using the HAD_50_ and RT-qPCR assays. As shown in Figures 2G, H, 100 µM DHM reduced ASFV progeny virus titers and ASFV-B646L transcription at every time point. Moreover, the inhibitory effect of DHM on the ASFV was stronger than that of the positive control genistein. These results indicate that DHM inhibited ASFV replication in a dose- and time-dependent manner. Furthermore, DHM inhibited PRRSV (NADC-30) and SIV (H1N1) replication in a dose-dependent manner, which indicates that DHM exhibits broad-spectrum antiviral effects (Additional file 2).

### DHM inhibited ASFV replication in the pre-, co-, and post-treatment modes

**Figure 3:**
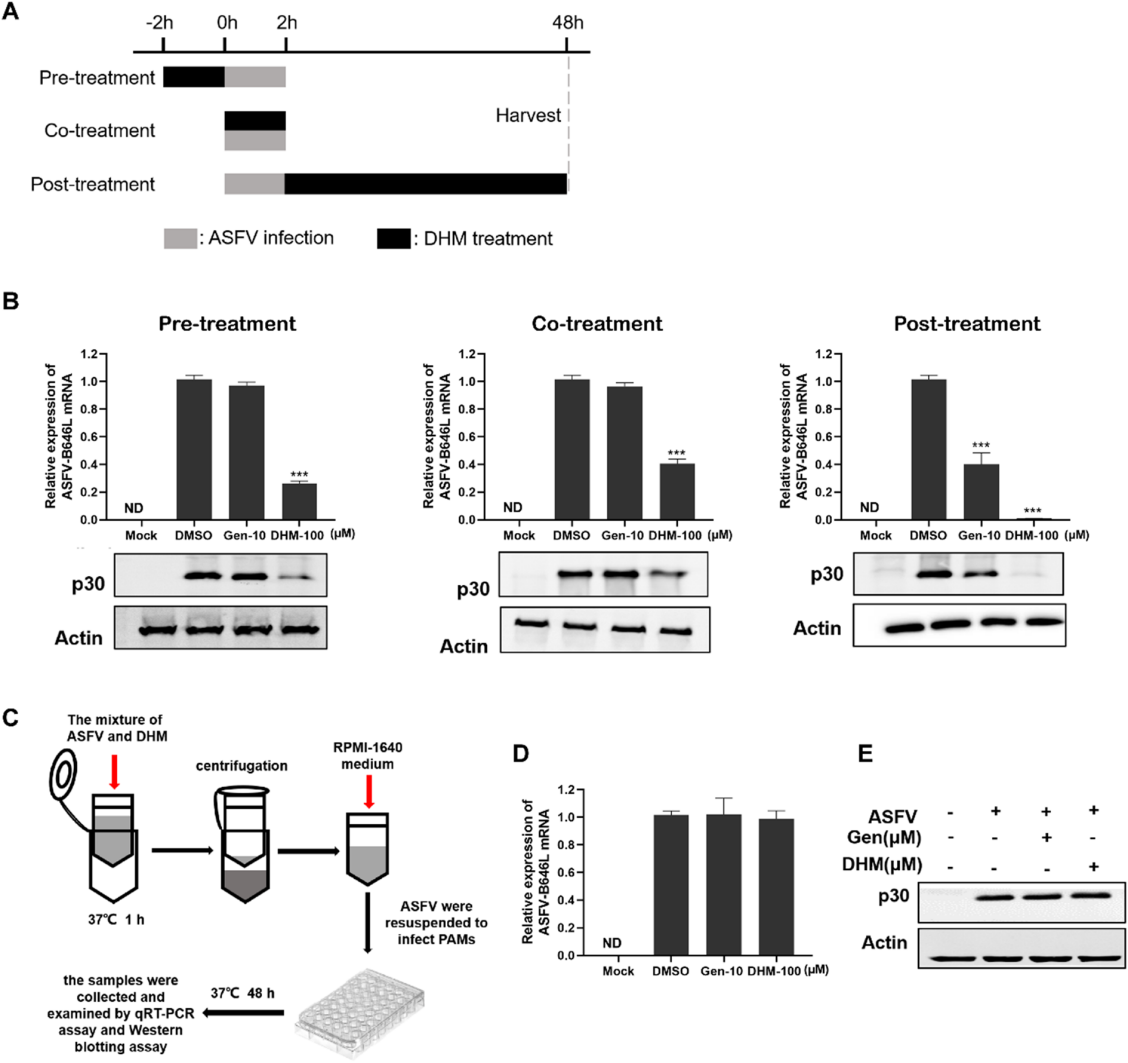
**DHM inhibited ASFV replication in various treatment modes in the time-to-addition assay.** **A** The schematic diagram of time-of-addition assays. **B** PAMs were treated with DHM before (pre-treatment), during (co-treatment), or after (post-treatment) infection with ASFV (1 MOI). The samples were further incubated for 48 h after ASFV infection, collected, and evaluated via Western blotting and RT-qPCR assay. **C** The schematic diagram of the direct interaction between ASFV and DHM. ASFV solution (1 MOI) was mixed with DHM for 1 h, and PAMs was treated with the ASFV separated using ultrafiltration tubes. After 48 h, the samples were collected for **D** Western blotting and **E** RT-qPCR assay. *P < 0.05, **P < 0.01, and ***P < 0.001 compared to the respective viral control.

We designed and performed time-of-addition assays (Figure 3A) with different treatment modes to explore how DHM inhibits ASFV replication. DHM was found to suppress ASFV infection in all modes (Figure 3B). Although DHM seemed to eliminate the ASFV in the co-treatment mode, we further designed and performed an assay for examining the direct interaction between the ASFV and DHM. The results demonstrated that DHM had no virucidal activity against ASFV (Figures 3D and E) and showed that DHM treatment inhibited ASFV replication by regulating cellular signaling pathways.

### DHM downregulated the ASFV-induced TLR4/MyD88 signaling

**Figure 4:**
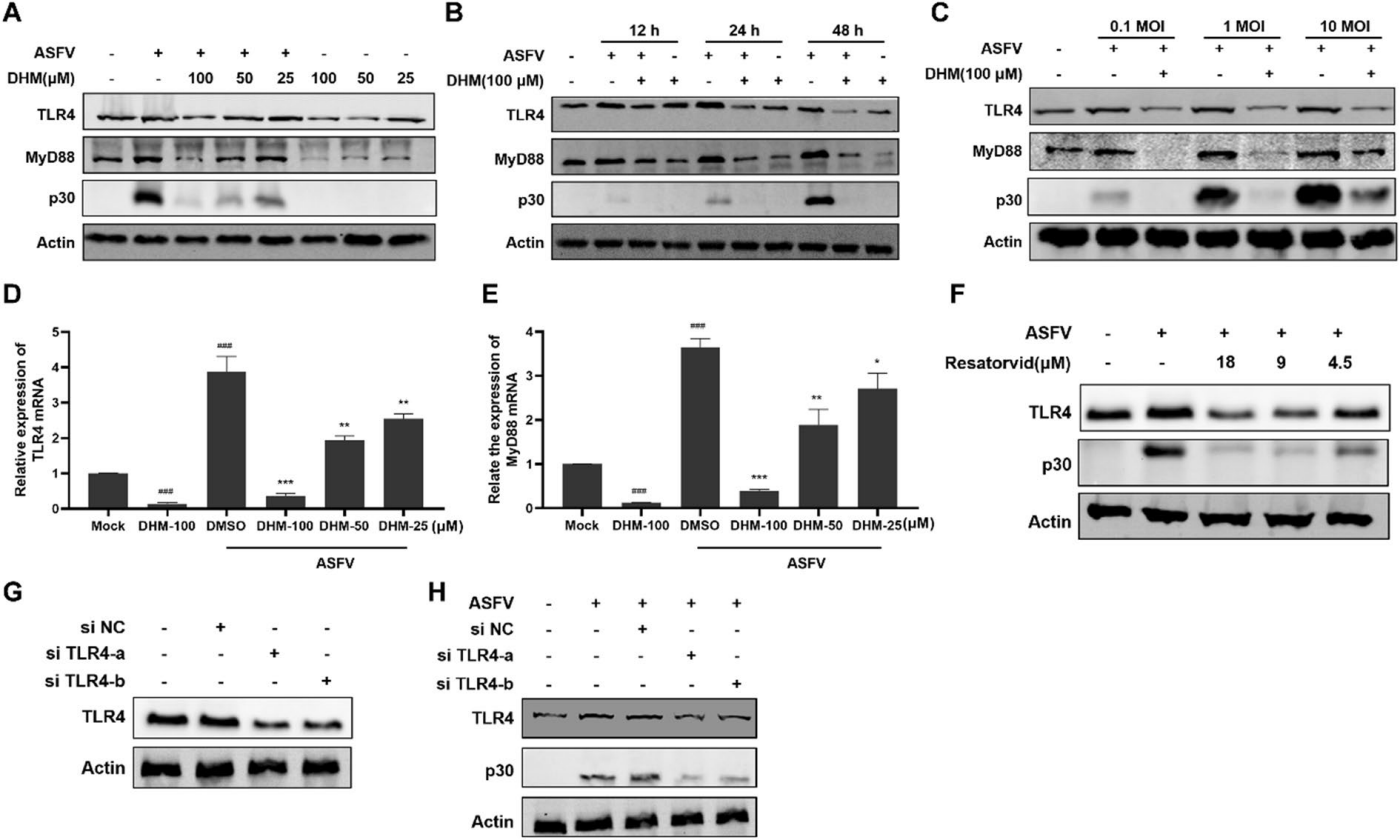
**DHM inhibited ASFV replication by regulating the TLR4/MyD88 signaling pathway.** **A** and **B** PAMs were incubated with ASFV solution (1 MOI) for 2 h before treatment with DHM. The samples were further incubated and collected at 24 h or at the indicated time points, and the samples were examined using Western blotting. **C** PAMs were infected with 0.1, 1, and 10 MOI ASFV solution for 2 h before treatment with DHM. Samples were collected after 24 h for Western blotting. **D** and **E** RT-qPCR of PAMs infected with ASFV solution before treatment with DHM; samples were collected 24 h after treatment. **F** Western blotting of ASFV-infected PAMs after a 24-h treatment with resatorvid. **G** Western blotting of PAMs transfected for 36 h with control siRNA (NC), siTLR4-a, or siTLR4-b. **H** PAMs were transfected with 50 nM siRNA control (NC), siTLR4-a, and siTLR4-b for 36 h. Next, the supernatants were removed, and PAMs were incubated with ASFV (1 MOI). After 24 h, the cells were collected and tested for Western blotting. *P < 0.05, **P < 0.01, and ***P < 0.001 compared to the respective virus control. ###*P* < 0.001 compared to the respective mock control.

Since ASFV-induced IL-1β expression is TLR4-dependent [18], we first investigated the expression levels of ASFV-induced TLR4 and MyD88 (a TLR4 signal transmitter) after DHM treatment. The results showed that ASFV infection upregulated TLR4 and MyD88, which were markedly downregulated upon treatment with DHM in a dose-dependent manner. Moreover, DHM treatment in uninfected cells also reduced TLR4 and MyD88 expression levels in a dose- and time-dependent manner (Figures 4A and B). Additionally, TLR4 and MyD88 expression levels increased with increasing ASFV infection doses (Figure 4C), which were, however, reduced by DHM treatment. Similarly, DHM reduced the ASFV-induced increased TLR4 and MyD88 mRNA expression levels (Figures 4D and E). Interestingly, we also found that the TLR4 inhibitor resatorvid (Additional file 3) suppressed ASFV replication and reduced ASFV-induced TLR4 expression (Figure 4F), suggesting that ASFV replication may be dependent on the TLR4 signaling pathway.

To further confirm the effect of TLR4 inhibition on ASFV, we designed siRNAs targeting TLR4. Treatment with siTLR4-a and siTLR4-b significantly decreased TLR4 expression compared to the control siRNA (NC) group (Figure 4G). Moreover, treatment with siTLR4-a and siTLR4-b before ASFV infection reduced p30 expression levels, indicating that TLR4 inhibition blocks ASFV replication cycle (Figure 4H). These results indicate that DHM exerts antiviral effects against ASFV by reducing the expression of TLR4, which is dependent on ASFV replication.

### DHM inhibited ASFV-induced inflammation by regulating MAPK/NF-κB signaling

**Figure 5:**
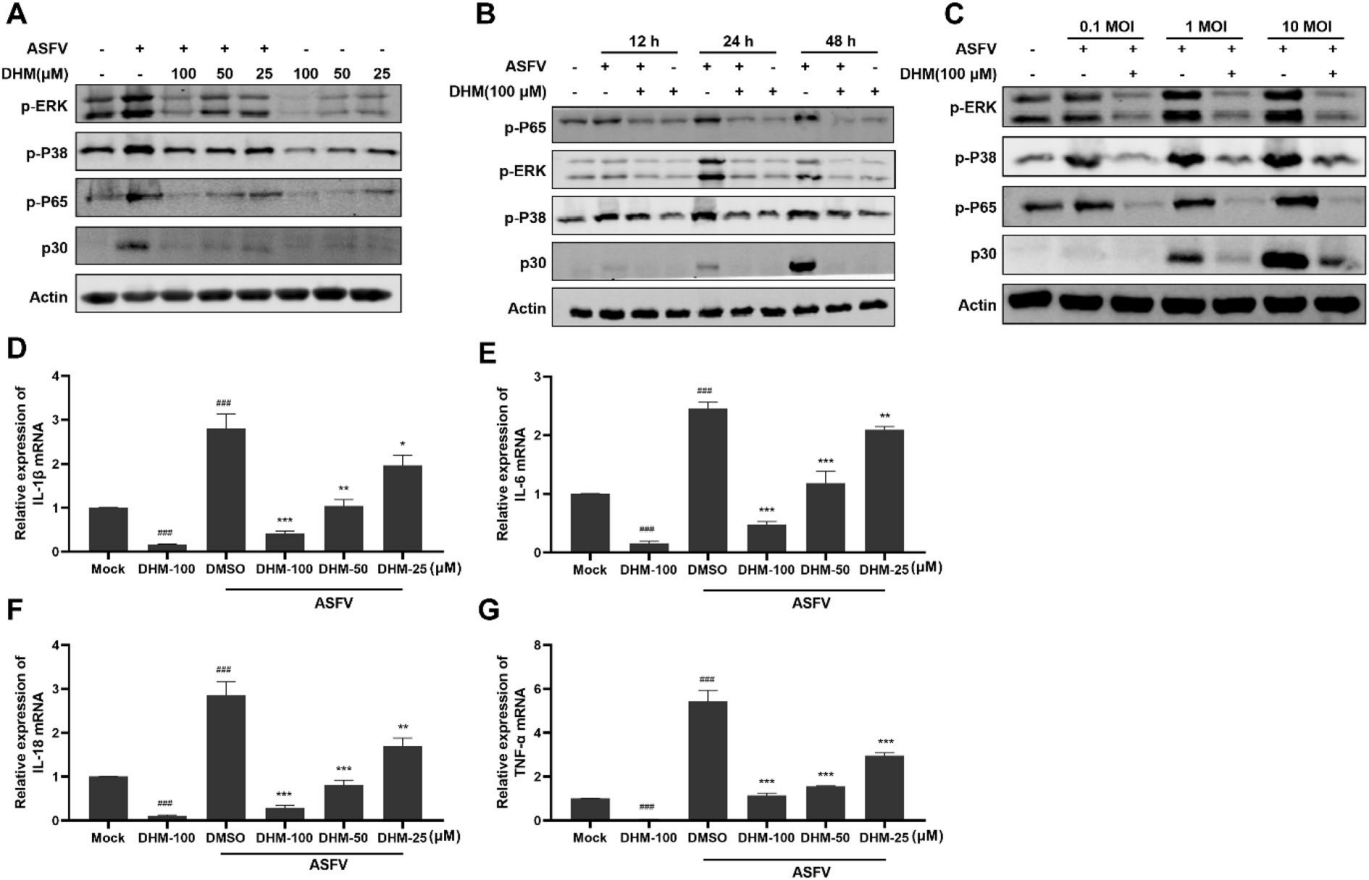
**DHM treatment downregulated ASFV-induced inflammatory mediators via the MAPK/NF-κB signaling pathway.** **A** and **B** Western blotting of ASFV-infected PAMs treated with DHM; samples were collected at 24 h of treatment or at the indicated time points. **C** Western blotting of PAMs infected with 0.1, 1, or 10 MOI ASFV solution before adding DHM; samples were collected after 24 h of treatment. **D**–**G** RT-qPCR of ASFV-infected PAMs treated with DHM; samples were collected at 24 h of treatment or at the indicated time points. *P < 0.05, **P < 0.01, and ***P < 0.001 compared to the respective viral control. ###*P* < 0.001 < 0.001 compared to the respective mock control.

TLR4 activates the MAPK/NF-κB signaling pathway in a MyD88-dependent manner, inducing the expression of TNF-α, IL-1β, IL-6, and IL-18, which are all end-effectors of the TLR4/MyD88/MAPK/NF-κB signaling pathway [31, 32]. Moreover, ASFV infection induces a cytokine storm in domestic pigs, leading to the overexpression of inflammatory cytokines, including TNF-α, IL-1β, IL-6, and IL-18 [33]. Therefore, we hypothesized that ASFV induces an excessive expression of inflammatory factors through the TLR4/MyD88/MAPK/NF-κB signaling pathway. First, we explored the effect of ASFV infection on the MAPK/NF-κB signaling pathway. The MAPK signaling pathway mainly comprises three subfamilies, including P38, ERK, and JNK [34]. However, we did not find suitable anti-JNK or anti-p-JNK antibodies for PAMs. ASFV infection increased phosphorylated P38, ERK, and p65 expression levels, which DHM then decreased in dose- and time-dependent (Figures 5A and B). Moreover, phosphorylated P38, ERK, and p65 expression levels increased with increasing ASFV doses but were reduced by DHM (Figure 5C). Consistent with previous results, ASFV infection promoted the expression of IL-1β, IL-6, IL-18, and TNF-α (Figures 5D-G), which DHM decreased in a dose-dependent manner. These results demonstrate that DHM reduces ASVF-induced inflammatory cytokine levels via the TLR4/MyD88/MAPK/NF-κB signaling pathway, thereby inhibiting ASFV replication.

### DHM treatment inhibited NLRP3 inflammasome activation

**Figure 6:**
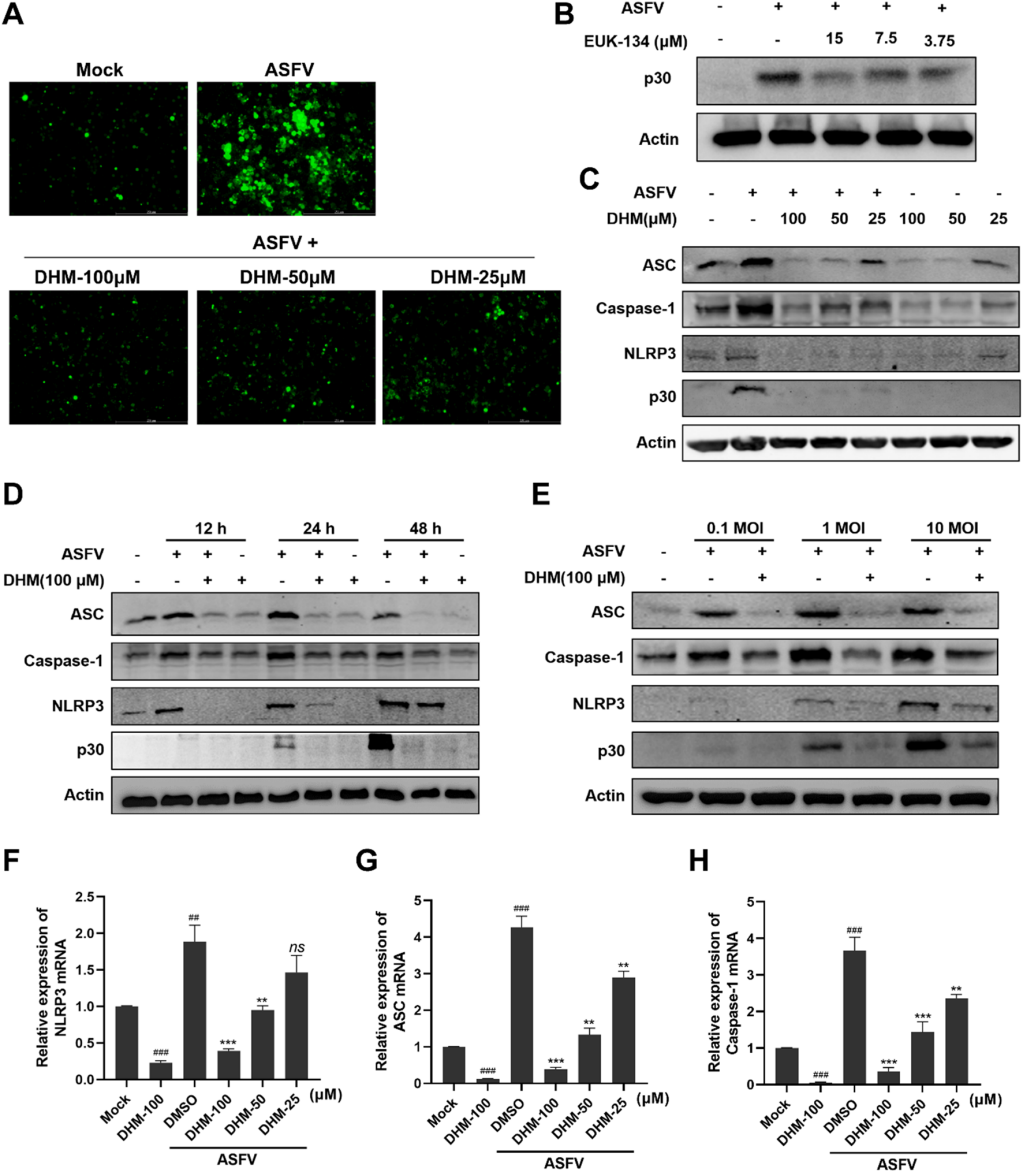
**DHM inhibited ASFV replication by inhibiting activation of the NLRP3 inflammasome.** **A** Fluorescent images of ASFV-infected PAMs stained with DCFH-DA after a 24-h treatment with DHM. **B**–**D** Western blotting of PAMs were treated with different concentrations of EUK-134 or with DHM after ASFV infection; samples were collected after 24 h of treatment or at the indicated time points. **E** Western blotting of PAMs infected with 0.1, 1, or 10 MOI ASFV solution before DHM treatment; samples were collected after 24 h of treatment. **F–G** RT-qPCR of ASFV-infected PAMs treated with DHM for 24 h. *P < 0.05, **P < 0.01, and ***P < 0.001 compared to the respective viral control. ###*P* < 0.001 compared to the respective mock control.

IL-1β and IL-18 significantly affect inflammation and immune responses [35]. NLRP3, ASC (apoptosis-associated speck-like protein), and pro-caspase-1 form the NLRP3 inflammasome when cells are infected with microorganisms or under certain stimuli. ASC recruited by NLRP3 interacts with pro-caspase-1 to form caspase-1, which transforms pro-IL-1β and pro-IL-18 into their active forms, IL-1β and IL-18, respectively [36]. Moreover, ROS are key in triggering NLRP3 inflammasome formation [37]. As ASFV infection stimulates IL-1β and IL-18 expression, we investigated the effect of ASFV infection on ROS production. We found that ASFV infection induced ROS accumulation, whereas DHM treatment reduced it (Figure 6A). Moreover, the antioxidant EUK-134 (Additional file 3) exerted an inhibitory effect on the ASFV (Figure 6B).

We then investigated the ASFV-induced changes in inflammasome component expression levels, namely NLRP3, ASC, and caspase-1. ASFV infection promoted their expression, but treatment with DHM reversed this increase in a dose- and time-dependent manner (Figures 6C and D) at different MOI (Figure 6E). Moreover, DHM treatment inhibited the expression of these proteins in uninfected PAMs. Similarly, DHM treatment also inhibited the expression of NLRP3, ASC, and caspase-1 mRNA (Figures 6F and G). These results suggest that DHM reduce ASFV-induced NLRP3 inflammasome activation.

### DHM treatment inhibited ASFV-induced pyroptosis

**Figure 7:**
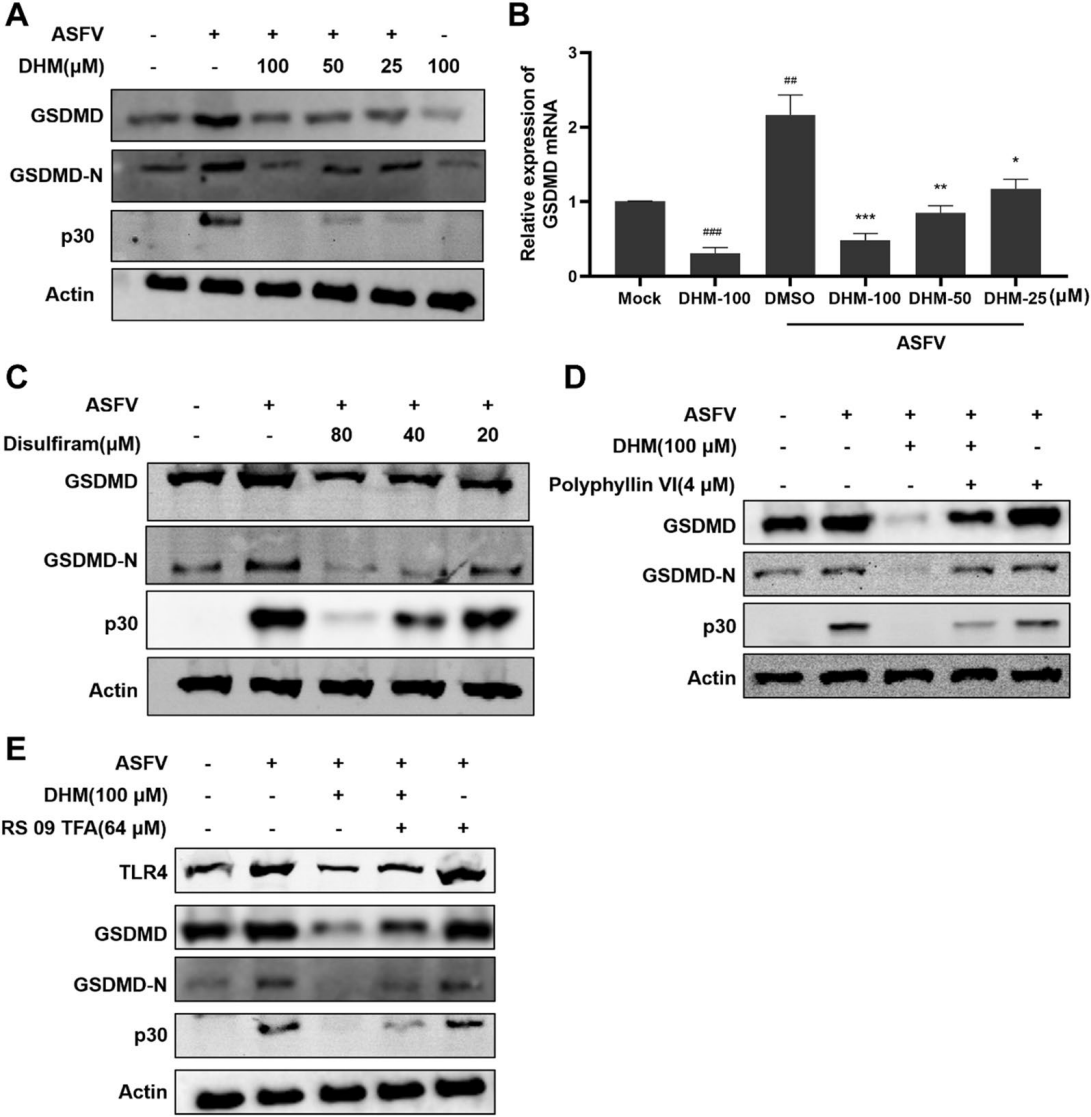
**DHM treatment inhibited ASFV-induced pyroptosis.** PAMs were treated with **A** and **B** DHM or **C** disulfiram after ASFV infection; the cells were then collected at 24 h for Western blotting or RT-qPCR. **D** and **E** Western blotting of ASFV-infected PAMs were treated with DHM in the presence or absence of **D** polyphyllin VI or **E** RS 09 TFA; samples were collected after 24 h of treatment. *P < 0.05, **P < 0.01, and ***P < 0.001 compared to the respective viral control. ^###^*P* < 0.001 compared to the respective mock control.

Our results demonstrate that ASFV infection induces the expression of caspase-1, IL-18, and IL-1β, which are related to pyroptosis. Caspase-1 also cleaves gasdermin D (GSDMD) to form GSDMD-C and GSDMD-N; subsequently, GSDMD-N forms a transmembrane pore and releases IL-18 and IL-1β, resulting in cell death and inflammation [38]. Therefore, we evaluated whether DHM treatment changes the expression of GSDMD and GSDMD-N. Our results suggested that ASFV infection induced the expression of GSDMD and GSDMD-N and that this effect was reduced by DHM treatment (Figure 7A). Moreover, DHM treatment reduced GSDMD mRNA expression levels (Figure 7B). Disulfiram, a pyroptosis inhibitor, simultaneously inhibited ASFV replication and ASFV-induced pyroptosis (Figure 7C). Conversely, the pyroptosis agonist polyphyllin VI (Additional file 3) partially reversed the inhibition of GSDMD, GSDMD-N, and ASFV p30 expression caused by DHM treatment (Figure 7D). Intriguingly, we found that inhibition of TLR4, GSDMD, GSDMD-N, and ASFV p30 expression after DHM treatment was partially reversed by RS 09 TFA (Additional file 3), a TLR4 agonist (Figure 7E), suggesting that DHM inhibits ASFV-induced pyroptosis by regulating TLR4.

## Discussion

Although ASF was discovered over 100 years ago, effective control measures have yet to be developed. Therefore, novel strategies are necessary to control ASFV transmission. In this study, we found that DHM exhibited the most remarkable anti-ASFV activity among 102 compounds and that it acted in a dose- and time-dependent manner. Moreover, DHM inhibited PRRSV and SIV replication, exhibiting potential broad-spectrum antiviral effects. Moreover, we found that DHM treatment inhibited ASFV replication through various treatment modes rather than by directly interacting with the virus.

The TLR family is crucial in regulating innate and adaptive immunities. TLR4 is a single transmembrane protein that is normally activated by LPS. Moreover, TLR4 is activated by damage associated molecular patterns released by virus-infected cells, such as S100A9, or is directly activated by viral proteins [39, 40]. Persistent TLR activation promotes inflammation and supports the replication of multiple viruses [13, 41]. TLR4 signals trigger the expression of MyD88 and TRIF, which induce the production of inflammatory cytokines and the expression of type I interferons, respectively [31]. Interestingly, ASFV-induced IL-1β expression is TLR4-dependent, confirming that TLR4 is important in ASFV infection [18]. Accordingly, we first explored the effects of ASFV infection on TLR4 and MyD88 and found that ASFV infection led to the upregulation of TLR4 and MyD88. Moreover, DHM can suppress LPS-induced TLR4 expression [42]. Similarly, we found that DHM suppressed TLR4 and MyD88 expression in ASFV-infected cells. We also found that DHM suppressed TLR4 and MyD88 expression in uninfected cells, which suggests that DHM may inhibit ASFV infection by reducing TLR4 and MyD88 expression in the pre-treatment mode. Furthermore, the TLR4 inhibitor resatorvid inhibited the expression of TLR4 and p30, and a TLR4-specific siRNA inhibited p30 expression, which suggested that TLR4 inhibition causes impairment of ASFV replication. However, further studies are needed to determine whether the ASFV indirectly or directly activates TLR4 expression.

The crosstalk between the NF-κB and MAPK pathways is a key regulatory mechanism of inflammation and immunity [43, 44]. Wohnke et al. analyzed the differences in protein expression between the highly pathogenic ASFV strain Armenia 2008 and the moderately pathogenic strain Estonia 2014 after infection with PAMs, using proteomics, and found that both strains upregulated phosphorylated p38 after 6 h of infection; however, how ASFV regulates the MAPK signaling pathway remains unknown [45]. Another study found that ASFV induced IL-1β and IL-8 expression by activating the NF-κB signaling pathway and that inhibition of the NF-κB signaling pathway inhibited ASFV replication [46]. The MAPK/NF-κB pathway is downstream of the TLR4/MyD88 pathway and regulates the inflammation induced by LPS and viruses [47, 48]. In our study, ASFV infection activated the expression of phosphorylated p65, P38, and ERK, an effect reversed by DHM. Moreover, ASFV infection causes an inflammatory cytokine storm that induces the overexpression of multiple inflammatory cytokines [33]. Consistently, we found that ASFV upregulated IL-1β, IL-6, IL-18, and TNF-α, whereas DHM treatment suppressed the expression of ASFV-induced inflammatory factors. These results indicated that DHM suppressed ASFV replication by regulating the TLR4/MyD88/MAPK/NF-κB signaling axis and inhibiting the production of inflammatory factors.

ROS, as cellular messengers, play a critical role in regulating signaling pathways. Cells produce ROS under normal physiological conditions; however, during microbial infections or inflammation, cells produce excessive ROS, leading to adverse effects [49]. Recently, ASFV p17 was reported to induce endoplasmic reticulum stress and ROS expression, thereby inhibiting cell proliferation [50]. Consistent with these reports, we also found that ASFV infection induced ROS accumulation. Evidence has shown that ROS promote pyroptosis by inducing NLRP3 expression [51]. Reportedly, DHM treatment inhibits ROS accumulation, thereby inhibiting NLRP3 inflammasome activation [52, 53]. Herein, DHM reduced the ASFV-induced ROS production and NLRP3, ASC, and caspase-1 expression. Additionally, ASFV infection upregulated GSDMD and GSDMD-N, which was reduced upon DHM treatment. Moreover, disulfiram, a pyroptosis inhibitor, reduced the ASFV-induced GSDMD and GSDMD-N expression. The inhibition of ASFV-induced pyroptosis through DHM treatment was partially reversed by the pyroptosis agonist polyphyllin VI. Interestingly, polyphyllin VI also induces apoptosis and autophagy, and apoptosis and autophagy are also associated with ASFV replication, whereas DHM has an inhibitory effect on apoptosis and autophagy [54, 55]. Therefore, the question is, does polyphyllin VI also reverse the inhibitory effect of DHM on ASFV-induced apoptosis and autophagy, and thus inhibit ASFV replication? Notably, the inhibition of ASFV-induced pyroptosis and TLR4 via DHM was partially reversed by the TLR4 agonist RS 09 TFA, suggesting that DHM inhibits ASFV-induced pyroptosis by regulating TLR4.

Jo et al. reported that myricetin and myricitrin have potent anti-African swine fever virus protease activity [56]. Natural flavonoid compounds myricetin and DHM have a similar chemical structure, suggesting that DHM may inhibit ASFV replication by acting as an African swine fever virus protease inhibitor [27]. This reveals that DHM may act as inhibitor against ASFV through a variety of mechanisms, but further research is needed. DHM has been extensively studied in vivo; it reduces thrombus formation induced by FeCl_3_, and its effects on glycemic control and insulin sensitivity in patients with type 2 diabetes are currently being evaluated in phase II clinical trials (NCT03606694) [57]. The toxic effects of flavonoids are an important issue in clinical applications. The maximum tolerated oral dose of DHM in rats was 5 g/kg and no toxicity was observed [58]. In another study to assess the toxicity of DHM, no hepatotoxicity, nephrotoxicity or blood cell damage was observed [59]. These findings confirm that DHM is relatively safe, and DHM capsules are sold as a nutritional supplement in the USA [60]. However, the main drawbacks of DHM are its chemical instability and poor bioavailability, which are the main factors limiting its clinical use. At present, DHM may not be used clinically for anti-ASFV treatment, but DHM can be used as a nutritional supplement. If the bioavailability of DHM is improved, DHM may be considered as a candidate for the treatment of ASFV. Taken together, we have provided a scientific basis for further studies on their use as anti-ASFV drugs in vivo.

**Figure 8:**
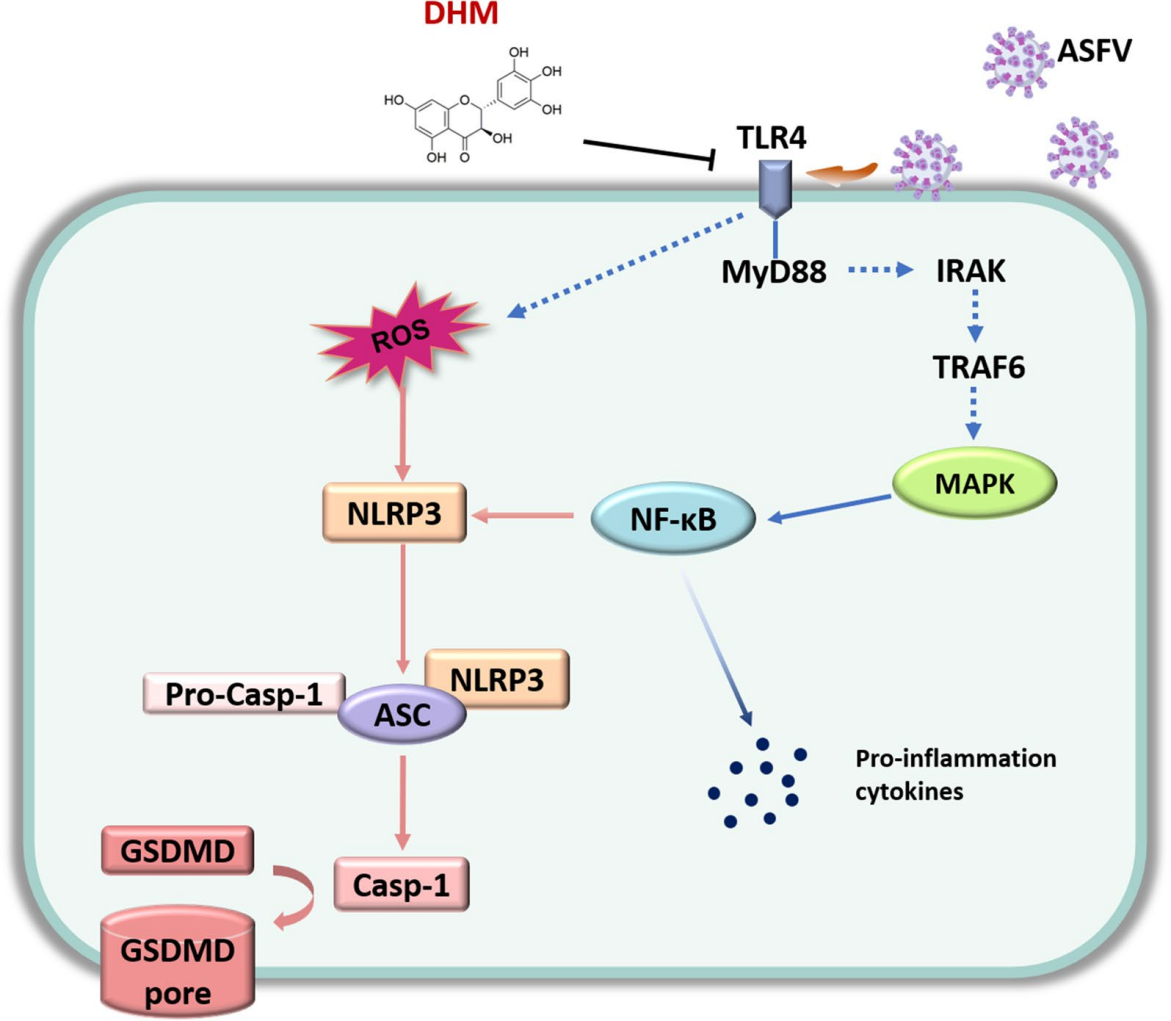
**Schematic model of the mechanism underlying DHM-mediated inhibition of on ASFV replication.** ASFV infection induces the expression of pro-inflammatory cytokines by activating the TLR4/MyD88/MAPK/NF-κB signaling pathway. Concomitantly, ASFV infection activates the ROS-NLRP3 inflammasome signaling cascade to induce pyroptosis. However, DHM treatment downregulates these signaling cascades and inhibits ASFV replication.

In summary, DHM inhibited ASFV replication in a dose- and time-dependent manner. Moreover, DHM treatment inhibited PRRSV and SIV replication, confirming that DHM possesses broad-spectrum antiviral activity. Mechanistic studies showed that DHM inhibited ASFV replication by reducing the levels of ASFV-induced inflammatory mediators via regulating the TLR4/MyD88/MAPK/NF-κB signaling pathway and suppressing pyroptosis. A schematic model of the inhibitory mechanism on ASFV replication of DHM is shown in Figure 8. Our results provide an experimental basis for studying novel candidate compounds for anti-ASFV drug development.

## Supplementary Information

**Additional file 1. DHM treatment reduced ASFV-induced hemadsorption**. PAMs were attached to the plates and infected with 1 MOI ASFV solution. After 2 h, the supernatants were removed, and PAMs were treated with a fresh medium containing DHM. After 24 h, 1% red blood cells of pig were added into per well. After 24 h of incubation, the samples were examined using a Leica DMI 4000 B fluorescence microscope (Leica, Wetzlar, Germany). (Black arrow indicates PAMs, red arrow indicates red blood cells of pig, and blue arrow indicates hemadsorption).

**Additional file 2. Antiviral activity of DHM againstPRRSV and SIV**. PAMs were attached to the plates and infected with 1 MOI **(A)** PRRSV or** (B)** SIV solution. After 2 h, the supernatants were removed, and PAMs were treated with fresh medium containing DHM. After 48 h, samples were collected for Western blotting.

**Additional file 3. Cytotoxicity of resatorvid, EUK-134, disulfiram, polyphyllin,and RS 09 TFA toward PAMs.** The effects of resatorvid, EUK-134, disulfiram, polyphyllin, and RS 09 TFA on the cellular viability of PAMs were examined using the cell-counting kit (CCK)-8 kit after incubation for 48 h. **p < 0.01 and ***p < 0.001 compared to the respective virus control.
