# Supplementary material for: Dihydromyricetin inhibits African swine fever virus replication by downregulating toll-like receptor 4-dependent pyroptosis in vitro
Source: Vet Res. 2023 Jul 12;54:58. doi: 10.1186/s13567-023-01184-8 (PMC10337113; doi:10.1186/s13567-023-01184-8)
Supplement: Supplementary file 1 — Additional file 1. DHM treatment reduced ASFV-induced hemadsorption. PAMs were attached to the plates and infected with 1 MOI ASFV solution. After 2 h, the supernatants were removed, and PAMs were treated with a fresh medium containing DHM. After 24 h, 1% red blood cells of pig were added into per well. After 24 h of incubation, the samples were examined using a Leica DMI 4000 B fluorescence microscope (Leica, Wetzlar, Germany). (Black arrow indicates PAMs, red arrow indicates red blood cells of pig, and blue arrow indicates hemadsorption). [file 13567_2023_1184_MOESM1_ESM.docx]

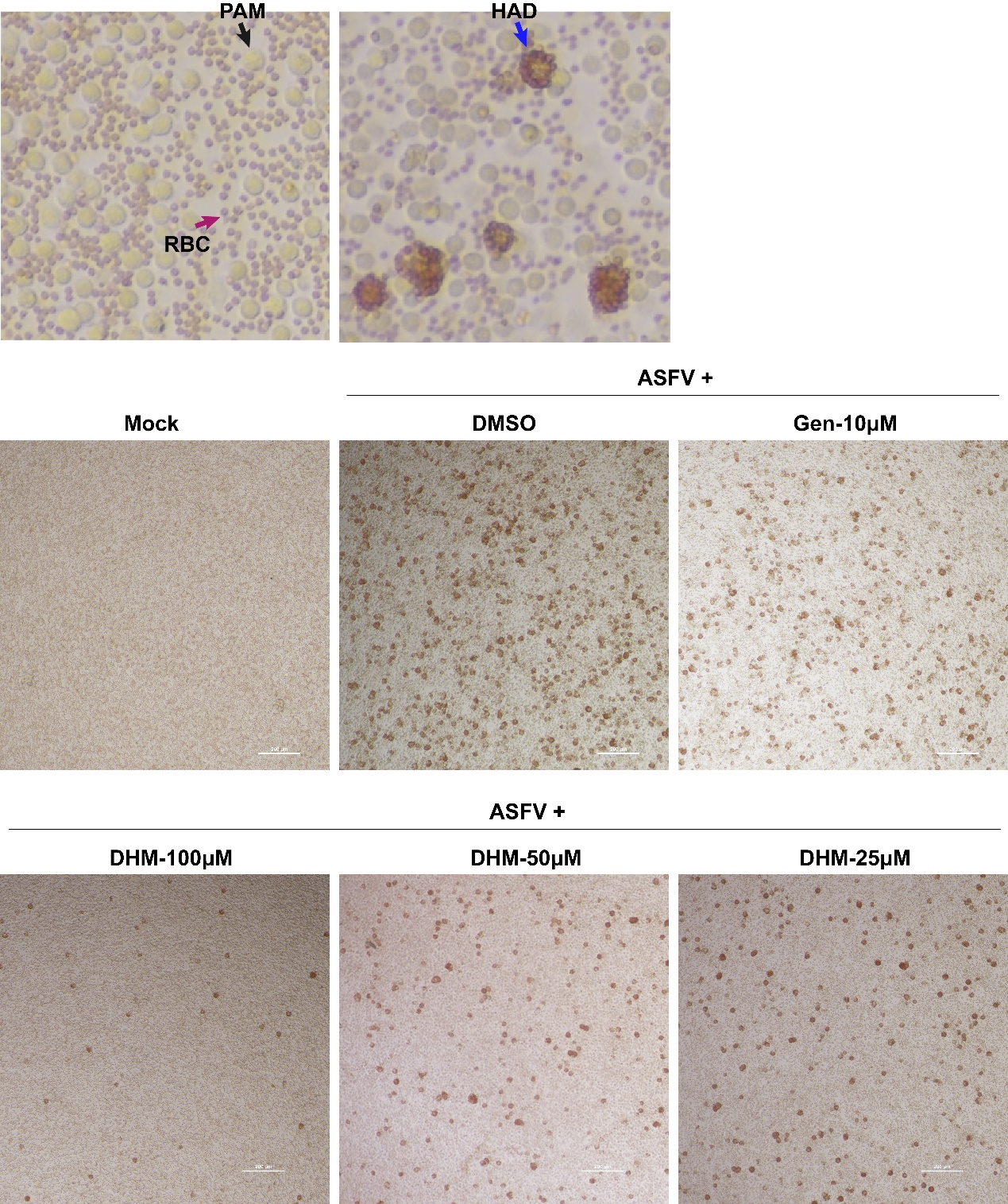


**Additional file 1.** **DHM treatment reduced ASFV-induced hemadsorption.** PAMs were attached to the plates and infected with 1 MOI ASFV solution. After 2 h, the supernatants were removed, and PAMs were treated with a fresh medium containing DHM. After 24 h, 1% red blood cells of pig were added into per well. After 24 h of incubation, the samples were examined using a Leica DMI 4000 B fluorescence microscope (Leica, Wetzlar, Germany). (Black arrow indicates PAMs, red arrow indicates red blood cells of pig, and blue arrow indicates hemadsorption).
