# Supplementary material for: Dihydromyricetin inhibits African swine fever virus replication by downregulating toll-like receptor 4-dependent pyroptosis in vitro
Source: Vet Res. 2023 Jul 12;54:58. doi: 10.1186/s13567-023-01184-8 (PMC10337113; doi:10.1186/s13567-023-01184-8)
Supplement: Supplementary file 2 — Additional file 2. Antiviral activity of DHM againstPRRSV and SIV. PAMs were attached to the plates and infected with 1 MOI (A) PRRSV or (B) SIV solution. After 2 h, the supernatants were removed, and PAMs were treated with fresh medium containing DHM. After 48 h, samples were collected for Western blotting. [file 13567_2023_1184_MOESM2_ESM.docx]

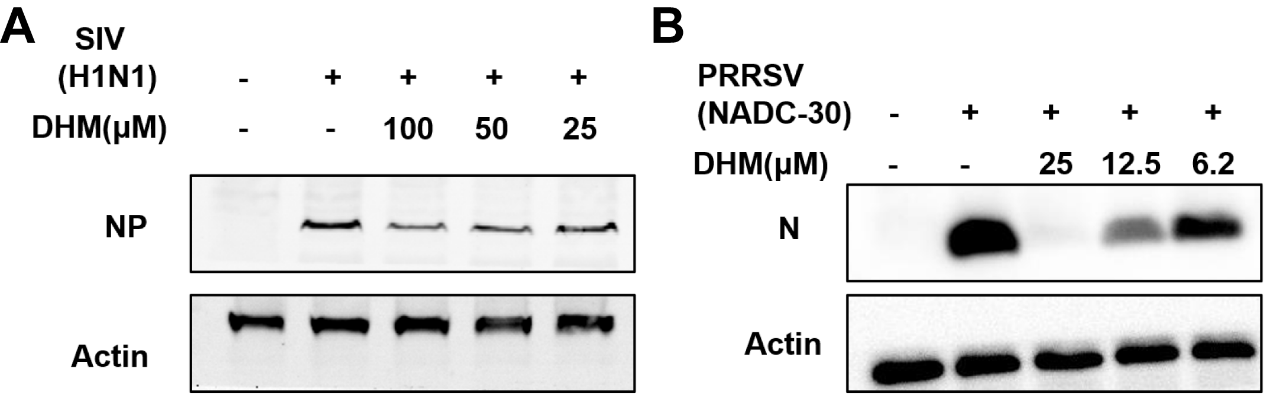


**Additional file 2. Antiviral activity of DHM against PRRSV and SIV.** PAMs were attached to the plates and infected with 1 MOI **(A)** PRRSV or **(B)** SIV solution. After 2 h, the supernatants were removed, and PAMs were treated with fresh medium containing DHM. After 48 h, samples were collected for Western blotting.
