# Supplementary material for: Dihydromyricetin inhibits African swine fever virus replication by downregulating toll-like receptor 4-dependent pyroptosis in vitro
Source: Vet Res. 2023 Jul 12;54:58. doi: 10.1186/s13567-023-01184-8 (PMC10337113; doi:10.1186/s13567-023-01184-8)
Supplement: Supplementary file 3 — Additional file 3. Cytotoxicity of resatorvid, EUK-134, disulfiram, polyphyllin,and RS 09 TFA toward PAMs. The effects of resatorvid, EUK-134, disulfiram, polyphyllin, and RS 09 TFA on the cellular viability of PAMs were examined using the cell-counting kit (CCK)-8 kit after incubation for 48 h. **p < 0.01 and ***p < 0.001 compared to the respective virus control. [file 13567_2023_1184_MOESM3_ESM.docx]

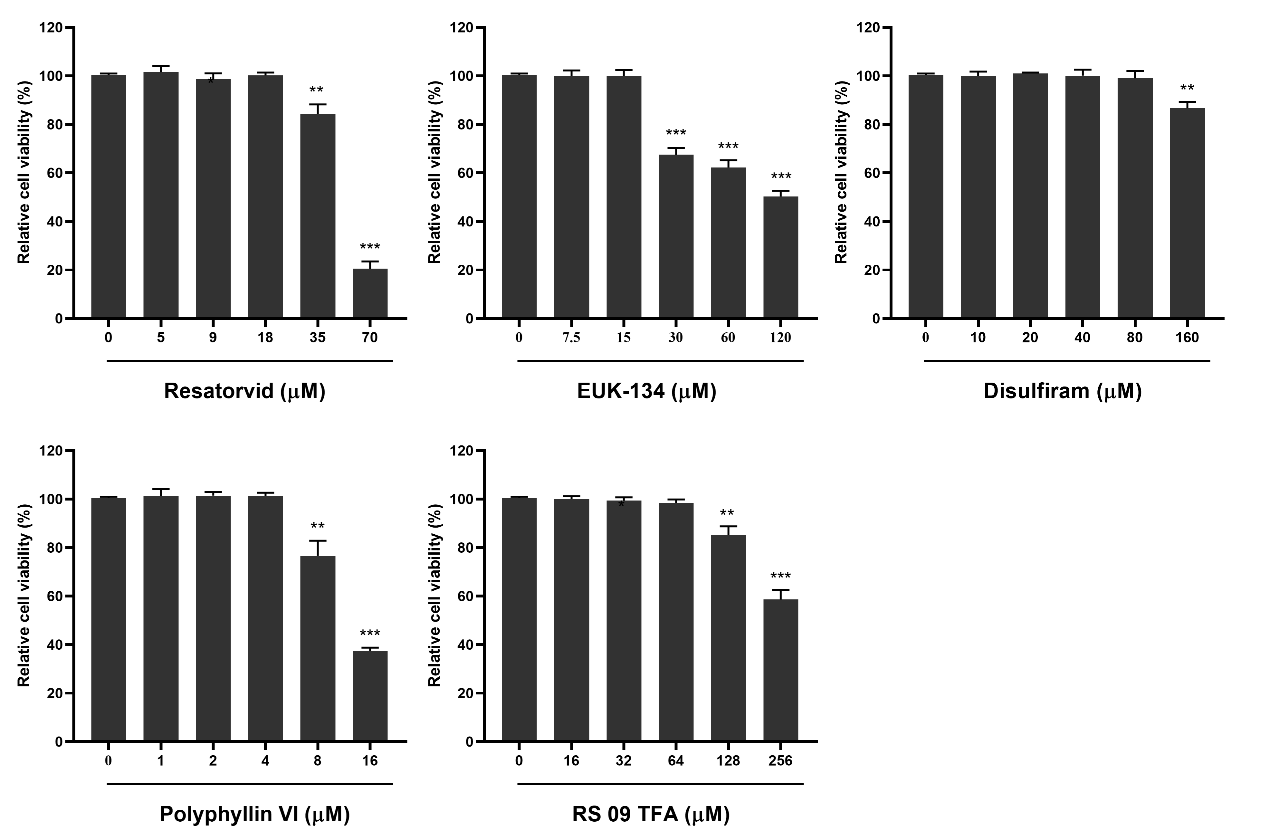


**Additional file 3. Cytotoxicity of resatorvid, EUK-134, disulfiram, polyphyllin, and RS 09 TFA toward PAMs.** The effects of resatorvid, EUK-134, disulfiram, polyphyllin, and RS 09 TFA on the cellular viability of PAMs were examined using the cell-counting kit (CCK)-8 kit after incubation for 48 h. ***p* < 0.01 and ****p* < 0.001 compared to the respective virus control.
